# Machine learning predicts mortality based on analysis of ventilation parameters of critically ill patients: multi-centre validation

**DOI:** 10.1186/s12911-021-01506-w

**Published:** 2021-05-07

**Authors:** Behrooz Mamandipoor, Fernando Frutos-Vivar, Oscar Peñuelas, Richard Rezar, Konstantinos Raymondos, Alfonso Muriel, Bin Du, Arnaud W. Thille, Fernando Ríos, Marco González, Lorenzo del-Sorbo, Maria del Carmen Marín, Bruno Valle Pinheiro, Marco Antonio Soares, Nicolas Nin, Salvatore M. Maggiore, Andrew Bersten, Malte Kelm, Raphael Romano Bruno, Pravin Amin, Nahit Cakar, Gee Young Suh, Fekri Abroug, Manuel Jibaja, Dimitros Matamis, Amine Ali Zeggwagh, Yuda Sutherasan, Antonio Anzueto, Bernhard Wernly, Andrés Esteban, Christian Jung, Venet Osmani

**Affiliations:** 1 Fondazione Bruno Kessler Research Institute, Trento, Italy; 2 Hospital Universitario de Getafe & Centro de Investigación en Red de Enfermedades Respiratorias (CIBERES), Madrid, Spain; 3 Clinic of Internal Medicine II, Department of Cardiology, Paracelsus Medical University of Salzburg, 5020 Salzburg, Austria; 4 Medizinische Hochschule Hannover, Hannover, Germany; 5 Unidad de Bioestadística Clinica Hospital Ramón y Cajal, Instituto Ramón y Cajal de Investigaciones Sanitarias (IRYCIS) & Centro de Investigación en Red de Epidemiología y Salud Pública (CIBERESP), Madrid, Spain; 6 Peking Union Medical College Hospital, Beijing, People’s Republic of China; 7 University Hospital of Poitiers, Poitiers, France; 8 Hospital Nacional Alejandro Posadas, Buenos Aires, Argentina; 9 Clínica Medellín & Universidad Pontificia Bolivariana, Medellín, Colombia; 10 Interdepartmental Division of Critical Care Medicine, Toronto, ON Canada; 11 Hospital Regional 1° de Octubre, Instituto de Seguridad Y Servicios Sociales de Los Trabajadores del Estado (ISSSTE), México, DF México; 12 Pulmonary Research Laboratory, Federal University of Juiz de Fora, Juiz de Fora, Brazil; 13 Hospital Universitario Sao Jose, Belo Horizonte, Brazil; 14 Hospital Español, Montevideo, Uruguay; 15 Università Degli Studi G. d’Annunzio Chieti e Pescara, Chieti, Italy; 16 Department of Critical Care Medicine, Flinders University, Adelaide, South Australia Australia; 17 Division of Cardiology, Pulmonology and Vascular Medicine, Medical Faculty, University of Düsseldorf, Moorenstraße 5, 40225 Düsseldorf, Germany; 18 Bombay Hospital Institute of Medical Sciences, Mumbai, India; 19 Istanbul Faculty of Medicine, Istanbul, Turkey; 20 Department of Critical Care Medicine, Samsung Medical Center, Sungkyunkwan University School of Medicine, Seoul, South Korea; 21 Hospital Fattouma Bourguina, Monastir, Tunisia; 22 Hospital de Especialidades Eugenio Espejo, Quito, Ecuador; 23 Papageorgiou Hospital, Thessaloniki, Greece; 24 Centre Hospitalier Universitarie Ibn Sina - Mohammed V University, Rabat, Morocco; 25 Faculty of Medicine Ramathibodi Hospital, Mahidol University, Bangkok, Thailand; 26 South Texas Veterans Health Care System and University of Texas Health Science Center, San Antonio, TX USA

**Keywords:** Critical care medicine, Machine learning, ICU, Risk stratification, Mechanical ventilation

## Abstract

**Background:**

Mechanical Ventilation (MV) is a complex and central treatment process in the care of critically ill patients. It influences acid–base balance and can also cause prognostically relevant biotrauma by generating forces and liberating reactive oxygen species, negatively affecting outcomes. In this work we evaluate the use of a Recurrent Neural Network (RNN) modelling to predict outcomes of mechanically ventilated patients, using standard mechanical ventilation parameters.

**Methods:**

We performed our analysis on VENTILA dataset, an observational, prospective, international, multi-centre study, performed to investigate the effect of baseline characteristics and management changes over time on the all-cause mortality rate in mechanically ventilated patients in ICU. Our cohort includes 12,596 adult patients older than 18, associated with 12,755 distinct admissions in ICUs across 37 countries and receiving invasive and non-invasive mechanical ventilation. We carry out four different analysis. Initially we select typical mechanical ventilation parameters and evaluate the machine learning model on both, the overall cohort and a subgroup of patients admitted with respiratory disorders. Furthermore, we carry out sensitivity analysis to evaluate whether inclusion of variables related to the function of other organs, improve the predictive performance of the model for both the overall cohort as well as the subgroup of patients with respiratory disorders.

**Results:**

Predictive performance of RNN-based model was higher with Area Under the Receiver Operating Characteristic (ROC) Curve (AUC) of 0.72 (± 0.01) and Average Precision (AP) of 0.57 (± 0.01) in comparison to RF and LR for the overall patient dataset. Higher predictive performance was recorded in the subgroup of patients admitted with respiratory disorders with AUC of 0.75 (± 0.02) and AP of 0.65 (± 0.03). Inclusion of function of other organs further improved the performance to AUC of 0.79 (± 0.01) and AP 0.68 (± 0.02) for the overall patient dataset and AUC of 0.79 (± 0.01) and AP 0.72 (± 0.02) for the subgroup with respiratory disorders.

**Conclusion:**

The RNN-based model demonstrated better performance than RF and LR in patients in mechanical ventilation and its subgroup admitted with respiratory disorders. Clinical studies are needed to evaluate whether it impacts decision-making and patient outcomes.

*Trial registration*: NCT02731898 (https://clinicaltrials.gov/ct2/show/NCT02731898), prospectively registered on April 8, 2016.

## Background

In the field of medicine, the use of computer-based algorithms for aiding diagnostic as well as therapeutic decisions has become a highly popular matter of often controversial discussions, whereas the question whether Artificial Intelligence (AI) might replace physicians someday arises time and again [1]. Even though it seems unlikely that AI will ever fully replace professional health care workers, it is advantageous to use computing power to analyse "big data" for the benefit of the patients. To solve complex mathematical problems, Deep Learning (DL) methods based on recurrent neural networks are used nowadays, especially in problems with temporal dependencies. In RNN weighted input values get summated and repeatedly updated to generate an output which best reflects the outcome of interest [2]. Furthermore, a memory function is generated by recurrent feedback mechanisms. The Long Short-Term Memory model (LSTM) by Hochreiter and Schmidhuber solves complex tasks by a constant error flow (“constant error carousels”) within memory cells with an opening and closing gate function, thereby enabling a quasi-sustained short-term memory [3]. Since their introduction, RNNs and especially LSTMs have been used for various tasks like handwriting recognition or speech recognition and in diverse healthcare applications [4]. Machine Learning (ML) already influences daily life more than we might be aware and it is indispensable for the technology industry.

In critical care medicine, the concept of ML for analysing complex and often highly heterogeneous patient collectives seems reasonable under various circumstances [5]. Different studies have evaluated the use of ML for the treatment of sepsis, assessing patient prognosis and/or risk for prolonged clinical courses and several other applications [6]. Regarding assessment of patients on mechanical ventilation and/ or prognostication of ICU-patients by AI, various studies were conducted that demonstrated that ML can be used as a prognostication tool for ICU-mortality [7, 8]. Parreco et al. were able to reliably identify patients at risk for tracheostomy and prolonged MV in their study on 20,262 ICU stays out of the MIMIC-III database [9]. Chen et al. were able to detect ventilator-associated pneumonia in patients on MV by using ML for the analysis of sensor arrays on exhaled breath samples [10]. Different other studies with promising results have been conducted in this field, which makes a future use of ML in clinical daily routine on the ICU likely.

## Objectives

We aim to investigate performance of these methods in a multi-centre cohort of patients in mechanical ventilation. In this investigation we rely on the VENTILA study group, a prospective, observational, international multi-centre cohort study that enrols patients on mechanical ventilation during a 28-day follow-up period. It comprises a large patient collective, generating a large amount of data and consequently rendering it suitable for the use of machine-learning methods. Mechanical ventilation is a complex and central treatment process in the care of critically ill patients. Not only represents a key element for treating respiratory insufficiency, but also significantly influences acid–base balance and can also cause prognostically relevant biotrauma by generating forces and liberating reactive oxygen species [11, 12]. It therefore represents a general outcome-relevant process for ICU patients. We aimed to evaluate the use of a LSTM-based model on a subgroup of mechanically ventilated critically ill patients out of the VENTILA study group to predict the outcome by using six standard mechanical ventilation parameters in our model. We follow STROBE guidelines [13] for reporting observational studies and provide the checklist of prediction model development and validation as a supplementary material.

## Methods

### Setting and data sources

VENTILA cohort dataset is a combination of four observational, prospective, international multi-center studies [14–16], performed to investigate the effect of baseline characteristics and management changes over time on the all-cause mortality rate in mechanically ventilated patients in ICU. VENTILA cohort includes adult patients older than 18, admitted to ICU receiving invasive (endotracheal tube or tracheostomy) and non-invasive (bilevel positive airway pressure (BIPAP) or continuous positive airway pressure (CPAP) with nasal or facial mask) mechanical ventilation for at least 12 and 1 h, respectively. Data recorded for all the patients included basic demographics, cause of requiring mechanical ventilation, the occurrence of complications, ICU and hospital discharge outcome and length of stay. Furthermore, daily collected data, resulting in a single average value, are recorded for variables such as arterial gases, mechanical ventilation parameters and variables related to the function of other organs. All the patients in this cohort study were followed for mortality and length-of-stay outcomes during the period of receiving mechanical ventilation, ICU stay, up to 28 days over one month period in 1998, 2004, 2010, and 2016. Only the investigative group members and research coordinators at each site were aware of the purpose and the precise timing of the study.

We evaluate our method on a sub-sample of VENTILA dataset containing data associated with 12,755 distinct hospital admissions for 12,596 adult patients (aged 18 years or above) admitted during one-month sample periods (in 2004, 2010 and 2016) from participating ICUs across 37 countries.

### Study subjects

We retrospectively evaluated the model on the overall VENTILA dataset (n = 12,755) as well as a sub-group of VENTILA patients that were admitted with respiratory disorders, specifically COPD, Asthma, interstitial lung disorders, ARDS or Pneumonia (n = 2674). Mortality rate in this subgroup was 36% (n = 960), while the overall morality was 31% (n = 3935).

### Statistical analysis and variable selection

**Fig. 1:**
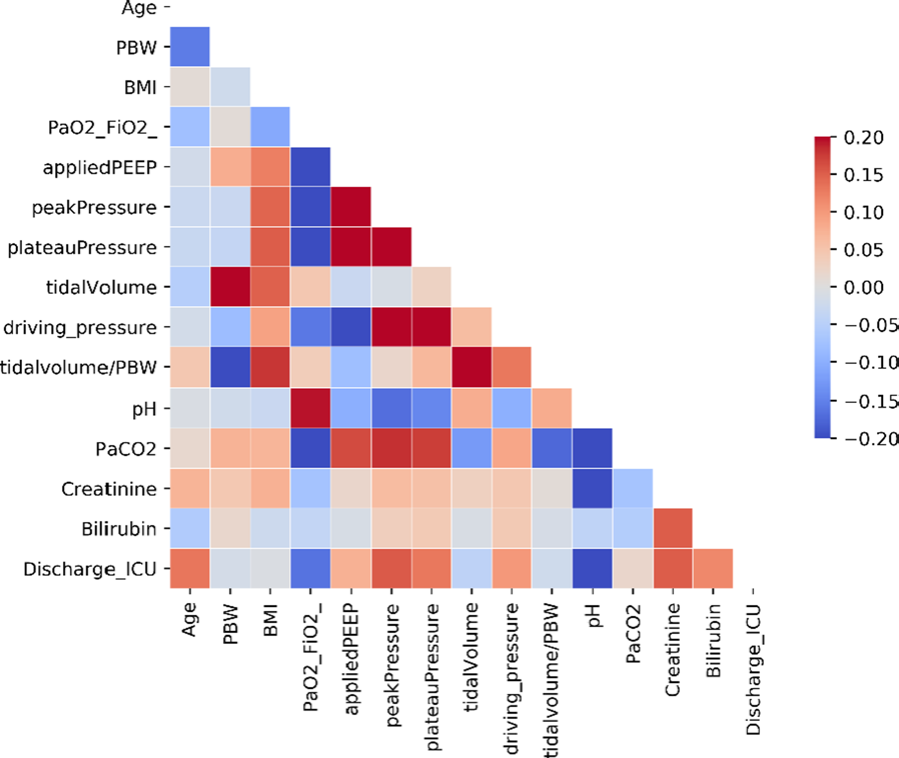
Linear correlation of variables and the outcome (indicated by Discharge ICU). Note the correlation scale is in the interval − 0.2 to 0.2

We use mean and standard deviation to express continuous variables, while categorical variables are expressed as a percentage. No strong linear correlations were found between input variables and the target outcome, as shown in Fig. 1.

We carry out four different analysis. Initially we selected six mechanical ventilation parameters as input to our model, namely PaO2_FiO2, peak Pressure, plateau Pressure, applied PEEP, driving pressure and tidal Volume/PBW, as well as age and BMI. We derive the model based on these variables for both: (1) patients in the overall dataset and (2) a subgroup of patients admitted with respiratory disorders.

Furthermore, we carry out a sensitivity analyses to evaluate whether inclusion of variables related to the function of other organs, such as such as kidneys (creatinine) and liver (bilirubin) improves the predictive performance of the model. This analysis is carried out for both: the overall dataset as well as on the subgroup of patients with respiratory disorders.

### Dataset pre-processing and missing values handling

Datasets were prepared for the analysis in several steps. Initially, outliers and noisy measurements were removed from the data by defining clinically valid intervals for each variable and considering out of interval values as missing values. Secondly, the Fill-Forward imputation methods were applied on each ICU-stay by forward propagating available values to use the nearest valid measurements. Since one of the most common reasons for missingness in ICU data is different frequency of measurements, using the nearest measured value becomes a suitable imputation strategy. Furthermore, imputation of variables completely missing during each ICU-stay was done using median of the variables in the training set. Finally, the data was normalized and scaled to have zero mean and unit variance such that variables with different scales can contribute equally to the analysis. The variable with the least missing values was ph (5.71%), while the highest was driving pressure (51.54%). The rest of the variables were as follows, PaCO2 (6.03%), appliedPEEP (7.97%), Creatinine (8.15%), PaO2_FiO2 (10.6%), tidalVolume (12.35%), tidalvolume/PBW (12.35%), peakPressure (17.4%), Bilirubin (25.94%), plateauPressure (51.33%).

### Predictive performance metrics

We assess the performance of our models using a range of performance metrics, including area under the ROC curve (AUC), area under the precision-recall curve (AUPRC) (also known as average precision) as well as positive predictive value (PPV), negative predictive value (NPV). We use Mathews correlation coefficient (MCC) [17] to compare quality of binary classifications between the different algorithms [18]. Lastly, we also investigate how well our model is calibrated, by plotting observed survival probability versus the survival probability predicted by our model using the calibration curve.

### Machine learning model

Our model uses ventilation parameters as an input to predict the likelihood of the patient dying. We restricted the analysis to include ventilation parameters only in order to evaluate their predictive power, while the rest of the variables from this dataset were excluded from the analysis. Patients were included if they had at the variables documented at least once, while the remaining patients were excluded.

In terms of machine learning algorithms we chose to evaluate Logistic Regression (LR) as the baseline model, Random Forest (RF), an ensemble of decision trees that has shown great performance in predicting clinical outcomes [19, 20] and the Long Short-Term Memory (LSTM) neural network [3], a type of Recurrent Neural Network (RNN). As RF and LR are unable to process sequences directly, we expanded each sequence into a single vector that was then fed to RF and LR. While there is some information loss in terms of timing of measurements, this approach attempts to minimise the loss, rendering the comparison as fair as possible between the algorithms.

The proposed LSTM network consist of one layer with 512 units, tanh activation function and Xavier normal weight initializer. Each patient record is classified in two possible outcomes using a SoftMax function in the output layer. We have also evaluated the Sigmoid function, but without discernible difference in the performance. Model derivation (training) is carried out for 150 epochs with batch size of 64 using binary cross entropy as loss function and Adam optimizer with learning rate 0.001. To ensure robustness and generalizability of the model we use a dropout layer with 0.5 and a custom L1 regularization layer with parameter of 0.0005. We use Dropout [21] to force the neural network to learn a more robust internal representation such that our model can generalise outcome predictions on data of future patients, while we use L1 regularisation method to reduce model complexity and susceptibility to overfitting, increasing generalisability.

The performance of each model was evaluated using stratified five-fold cross-validation with 10 times repetition. First, we split the data randomly into model derivation set (80%) and model validation set (20%). Then we built the model and tuned the hyper-parameters based on the validation set. We repeat five-fold cross-validation 10 times on the tuned model to reduce possible bias and evaluate generalisability, where for each run we calculate mean and standard deviation. The LSTM model is implemented using PyTorch [22] open source machine learning framework and we also used the scikit-learn software library for the non-RNN models implementation.

## Results

The overall dataset contained 12,755 ICU stays with complete data on ventilation parameters included in this study, where 3935 ICU stays were recorded as dead (30.85%). The respiratory disorders subgroup contained 2674 ICU stays with 960 ICU stays recorded as dead. The most common diagnosis for this subgroup was pneumonia (n = 1368) followed by COPD (n = 527), ARDS (n = 501), CPD_nonCOPD (n = 180) and asthma (n = 98). Survivors are compared to non-survivors for the overall patient dataset (Table 1(a)) as well as the subgroup of patients with respiratory disorders (Table 1(b)).

**Table 1:** (a) Baseline demographics of survivors versus non-survivors for all patients, (b) Baseline demographics of survivors versus non-survivors for patients admitted with respiratory disorders

Variables	Survivors	Non-survivors	p-value
*(a) Overall cohort*			
Female sex n (%)	3298 (37)	1452 (37)	
Age	58.87 ± 17.55	63.65 ± 16.16	< 0.01
Weight	75.36 ± 19.56	74.42 ± 19.32	0.01
PBW	62.17 ± 9.29	61.76 ± 9.31	0.02
BMI	26.56 ± 6.37	26.42 ± 6.29	0.25
Creatinine	1.38 ± 1.38	1.87 ± 1.66	< 0.01
Bilirubin	1.51 ± 3.39	2.55 ± 5.32	< 0.01
pH	7.40 ± 0.09	7.36 ± 0.12	< 0.01
PaCO2	39.96 ± 10.20	40.41 ± 11.81	0.15
PaO2_FiO2	257.99 ± 106.03	220.47 ± 107.66	< 0.01
peakPressure	23.98 ± 7.41	26.50 ± 7.89	< 0.01
plateauPressure	19.40 ± 5.65	21.03 ± 6.46	< 0.01
drivingPressure	12.64 ± 5.37	13.83 ± 6.13	< 0.01
appliedPEEP	6.66 ± 3.13	7.19 ± 3.60	< 0.01
tidalVolume	509.42 ± 118.72	498.82 ± 115.99	< 0.01
tidalvolume/PBW	8.30 ± 1.99	8.21 ± 2.13	< 0.01
SAPS_II	42.71 ± 17.04	55.08 ± 19.05	< 0.01
Propensity test	0.63 ± 0.03	0.63 ± 0.03	< 0.01
LOS in ICU	13.02 ± 13.45	11.70 ± 14.27	< 0.01
MV_days	8.41 ± 8.56	9.08 ± 10.10	< 0.01
*(b) Cohort admitted with respiratory disorders*			
Female sex n (%)	653 (38)	340 (35)	
Age	61.16 ± 17.19	63.94 ± 15.47	< 0.01
Weight	74.58 ± 23.13	71.80 ± 21.28	< 0.01
PBW	61.06 ± 9.33	61.32 ± 9.36	0.49
BMI	26.64 ± 7.80	25.58 ± 6.94	< 0.01
Creatinine	1.27 ± 1.14	1.75 ± 1.58	< 0.01
Bilirubin	1.44 ± 2.99	2.13 ± 4.66	0.01
pH	7.39 ± 0.09	7.35 ± 0.12	< 0.01
PaCO2	44.62 ± 13.13	45.14 ± 13.88	0.12
PaO2_FiO2	218.65 ± 94.10	178.84 ± 92.50	< 0.01
peakPressure	26.49 ± 8.13	29.07 ± 8.27	< 0.01
plateauPressure	21.41 ± 6.18	23.03 ± 6.75	< 0.01
drivingPressure	13.77 ± 5.80	14.70 ± 6.73	< 0.01
appliedPEEP	7.52 ± 3.77	8.27 ± 4.02	< 0.01
tidalVolume	478.62 ± 122.74	481.34 ± 121.99	< 0.01
tidalvolume/PBW	7.95 ± 2.15	7.97 ± 2.09	< 0.01
SAPS_II	43.37 ± 16.27	51.02 ± 18.30	< 0.01
Propensity test	0.64 ± 0.03	0.64 ± 0.03	< 0.01
LOS in ICU	15.72 ± 15.93	13.78 ± 15.74	< 0.01
MV_days	10.20 ± 10.51	10.48 ± 10.77	0.52

**Fig. 2:**
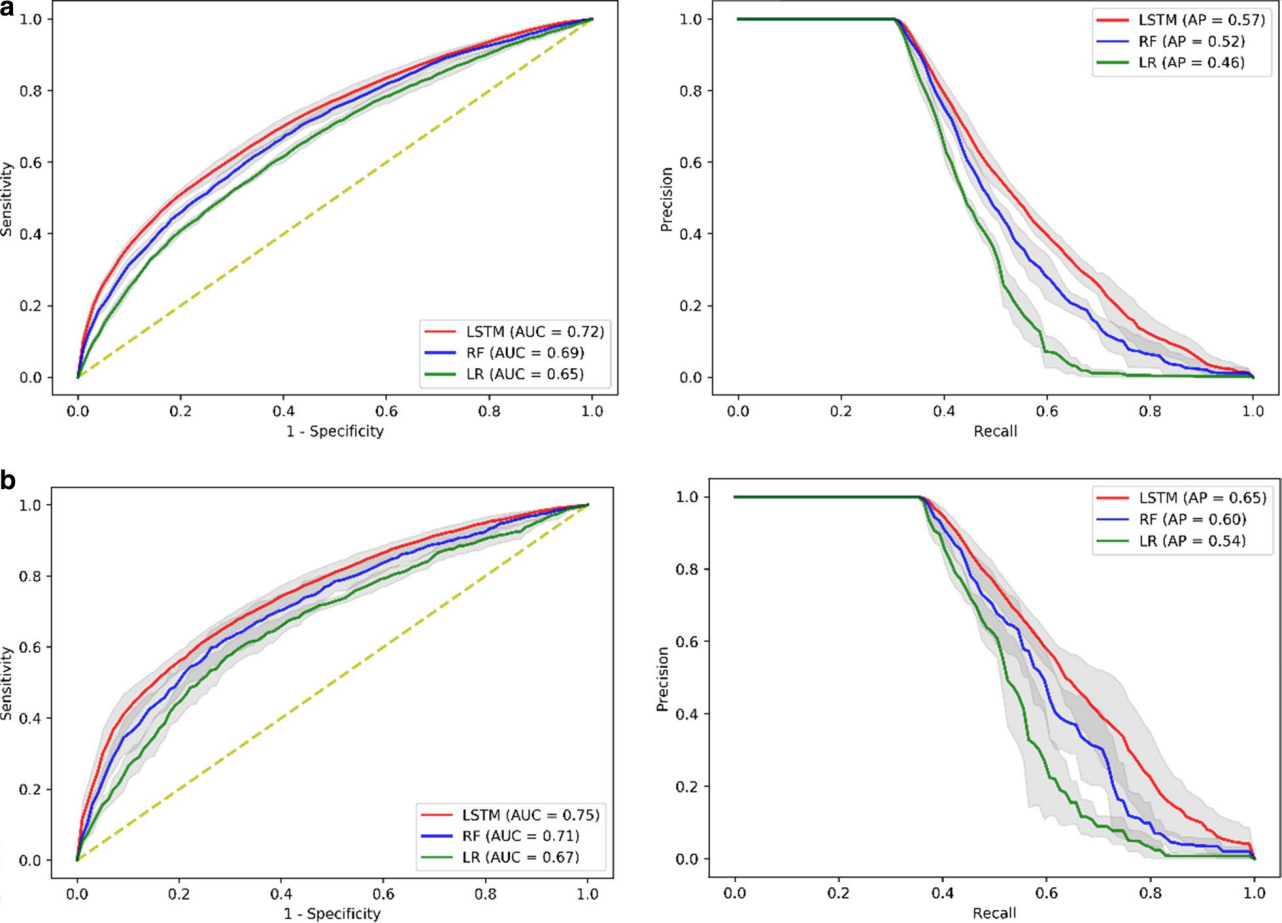
Panel **a**. Predictive performance (AUC and AUPRC) of our LSTM-based model versus Random Forrest (RF) and Logistic Regression (LR) for the overall patient dataset using six standard mechanical ventilation parameters. Panel **b**. Predictive performance of our LSTM-based model versus Random Forrest (RF) and Logistic Regression (LR) for the subgroup of patients admitted with respiratory disorders using six standard mechanical ventilation parameters. Confidence intervals are shown in grey for both panels

We compared our LSTM-based model with both random forrest and logistic regression methods. Predictive performance of LSTM-based model was higher with AUC of 0.72 and Average Precision (AP) of 0.57 in comparison to RF and LR for the overall patient dataset. Higher predictive performance of AUC of 0.75 and AP of 0.65 was recorded in the subgroup of patients admitted with respiratory disorders, as shown in Fig. 2a, b.

Other performance measures, such as PPV, NPV and MCC are detailed in Table 2(a) and (b), where LSTM-based model outperforms both RF and LR in the majority of performance metrics.

**Table 2:** (a) Performance of the models for the overall patient dataset using six standard mechanical ventilation parameters, (b) Performance of the models for the subgroup of patients admitted with respiratory disorders using six standard mechanical ventilation parameters

	AUC	AP	PPV	NPV	MCC
*(a) Overall cohort*					
LR	0.65 ± 0.01	0.46 ± 0.01	0.50 ± 0.02	0.74 ± 0.01	0.21 ± 0.01
RF	0.69 ± 0.01	0.52 ± 0.01	0.51 ± 0.02	0.76 ± 0.01	0.26 ± 0.01
LSTM	**0.72 ± 0.01**	**0.57 ± 0.01**	**0.52 ± 0.03**	**0.79 ± 0.01**	**0.31 ± 0.02**
*(b) Cohort admitted with respiratory disorders*					
LR	0.67 ± 0.02	0.54 ± 0.03	0.54 ± 0.03	0.74 ± 0.01	0.28 ± 0.03
RF	0.71 ± 0.02	0.60 ± 0.02	0.54 ± 0.04	0.76 ± 0.02	0.31 ± 0.06
LSTM	**0.75 ± 0.02**	**0.65 ± 0.03**	**0.59 ± 0.03**	**0.79 ± 0.01**	**0.37 ± 0.03**

Highest performance is shown in bold

### Sensitivity analysis

We also performed a sensitivity analysis to evaluate whether inclusion of function of other organs, such as kidney and liver could improve predictive performance of the model. This analysis was carried out for the overall dataset as well as for the subgroup of patients admitted with respiratory disorders, where we included creatinine and bilirubin variables as well as pH and PaCO2, in addition to the variables used for the main analysis. These variables were chosen based on the review of literature as well as suggestion from the clinicians.

**Fig. 3:**
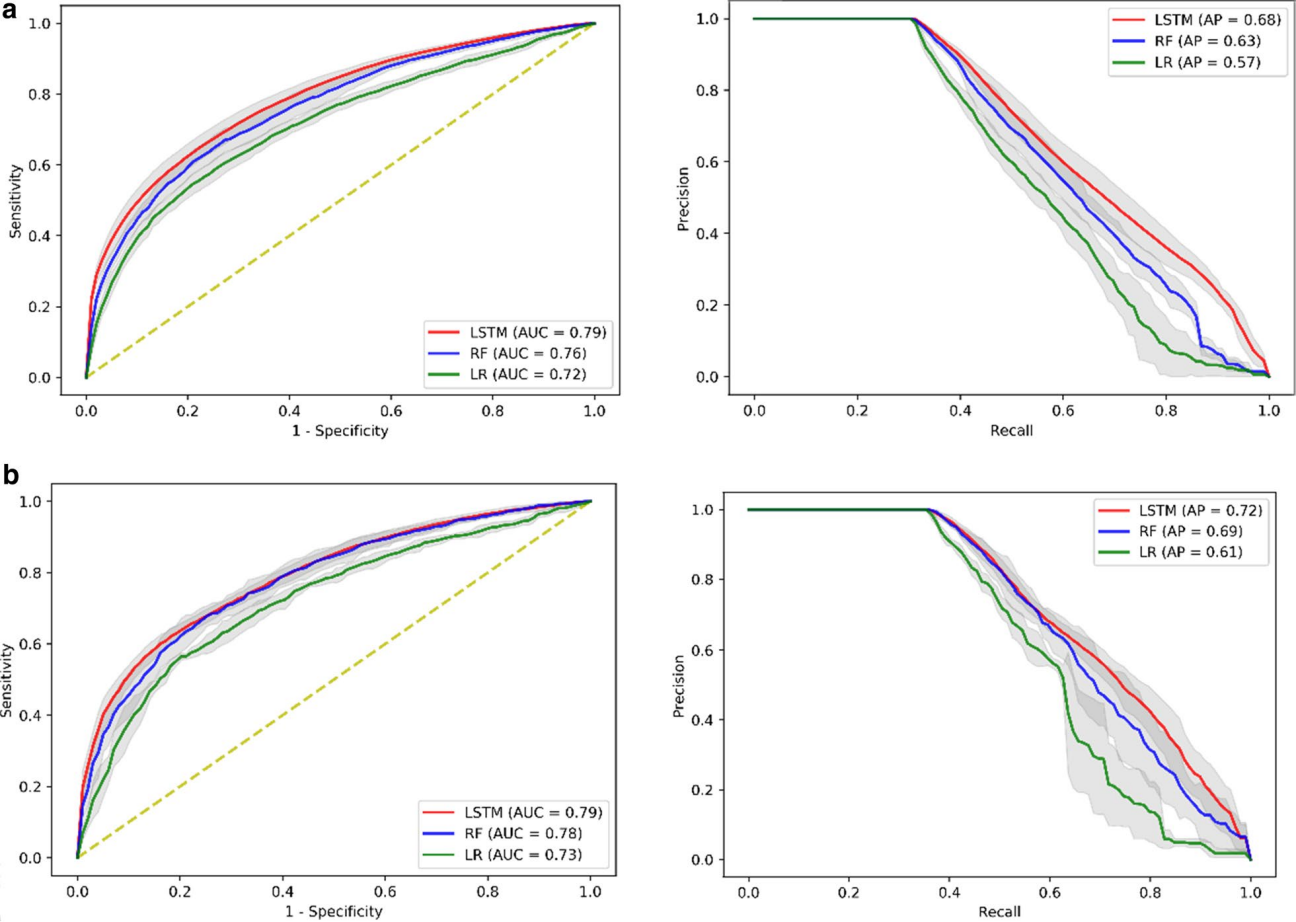
Panel **a**. Predictive performance (AUC and AUPRC) of our LSTM-based model versus Random Forrest (RF) and Logistic Regression (LR) for the overall patient dataset, including also variables related to kidney and liver function. Panel **b**. Predictive performance of our LSTM-based model versus Random Forrest (RF) and Logistic Regression (LR) for the subgroup of patients admitted with respiratory disorders, including also variables related to kidney and liver function. Confidence intervals are shown in grey for both panels

As it can be seen from Fig. 3a, b, the inclusion of variables related to kidney and liver function increased predictive performance significantly with AUC of 0.79 and AP 0.68 for the overall patient dataset and AUC of 0.79 and AP 0.72, for the subgroup of patients with respiratory disorders. As shown in Table 3(a) and (b) LSTM based model outperforms RF and LR in the majority of performance metrics.

**Table 3:** (a) Performance of the models for the overall patient dataset, by also including variables related to kidney and liver function, (b) Performance of the models for the subgroup of patients admitted with respiratory disorders, by also including variables related to kidney and liver function

	AUC	AP	PPV	NPV	MCC
*(a) Overall cohort*					
LR	0.72 ± 0.02	0.57 ± 0.03	0.58 ± 0.03	0.78 ± 0.01	0.34 ± 0.03
RF	0.76 ± 0.02	0.63 ± 0.02	**0.59 ± 0.04**	0.80 ± 0.01	0.38 ± 0.03
LSTM	**0.79 ± 0.02**	**0.68 ± 0.02**	**0.59 ± 0.04**	**0.83 ± 0.01**	**0.42 ± 0.04**
*(b) Cohort admitted with respiratory disorders*					
LR	0.73 ± 0.01	0.61 ± 0.01	0.58 ± 0.03	0.77 ± 0.02	0.35 ± 0.03
RF	**0.78 ± 0.02**	0.69 ± 0.04	0.61 ± 0.05	**0.80 ± 0.02**	0.41 ± 0.06
LSTM	**0.79 ± 0.01**	**0.72 ± 0.02**	**0.63 ± 0.04**	**0.80 ± 0.01**	**0.43 ± 0.03**

Highest performance is shown in bold

### Variable importance and model interpretability

**Fig. 4:**
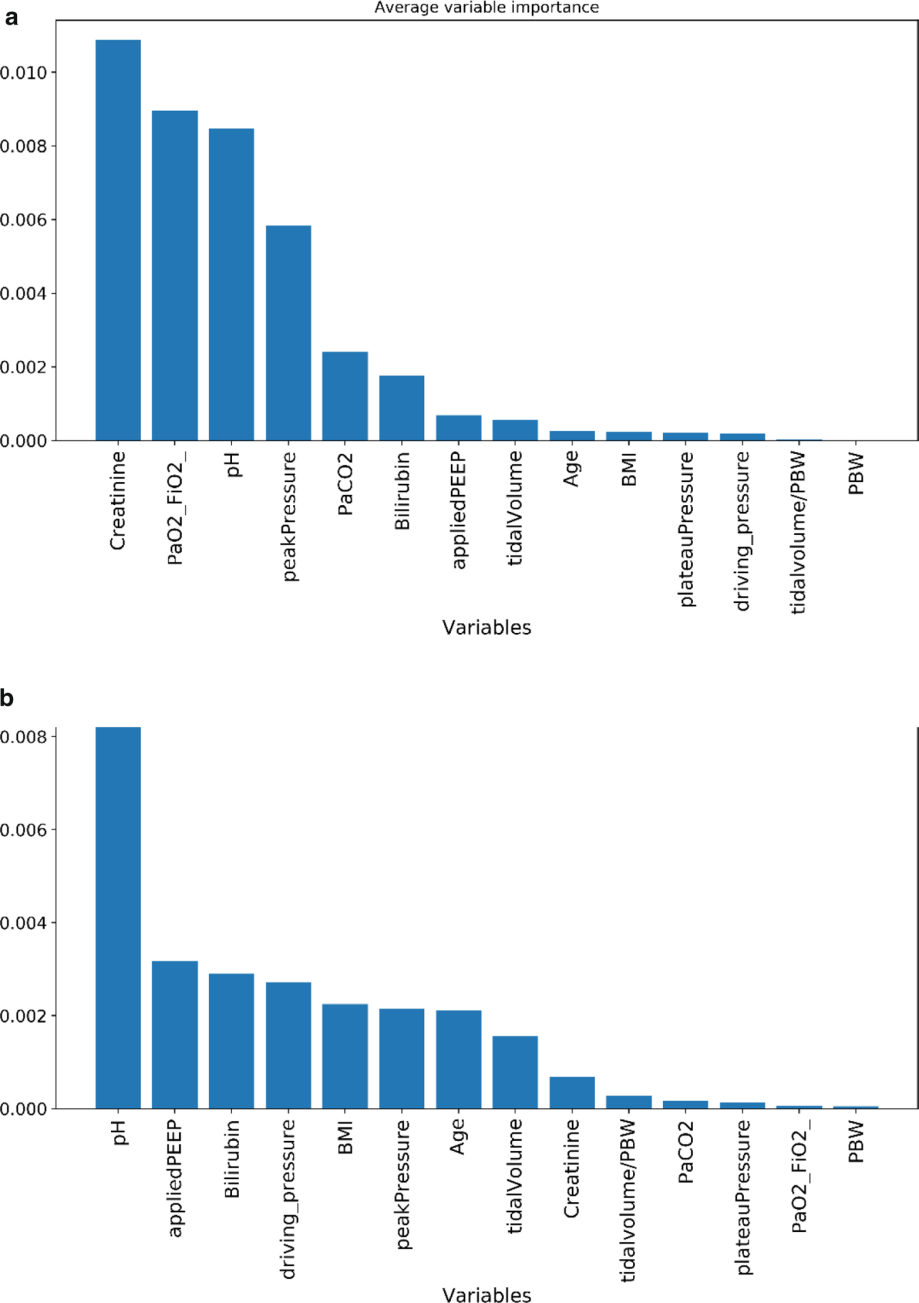
Variable importance ranking for each LSTM model: a) All patients, and b) patients admitted with respiratory disorders

A common criticism of LSTM-based models in particular and neural-network models in general, is that they are regarded as black-box models [23, 24]. We sought to address this issue by conducting a model interpretability analysis, to understand how the model ranked the importance of variables when predicting mortality outcomes. We used the Integrated Gradients (IG) method whose objective is to illustrate the relationship between a model’s prediction outcome and its’ input variables [25]. IG method explains outcomes of the LSTM models based on the gradients of the prediction outcomes with respect to input variables. By computing attribution of each variables, it ranks all the variables based on their importance. The attribution values measure the effect of each feature relative to the prediction for a baseline, which in our case was set to zero. As a result, the top three ranked variables of the LSTM model were creatinine, PaO2_FiO2, and pH (for the overall patient dataset) and pH, appliedPEEP, and Bilirubin (for the subgroup of patients with respiratory disorders). A graphical representation of the variables for each model and their ranking is provided in Fig. 4.

### Predictive model calibration

**Fig. 5:**
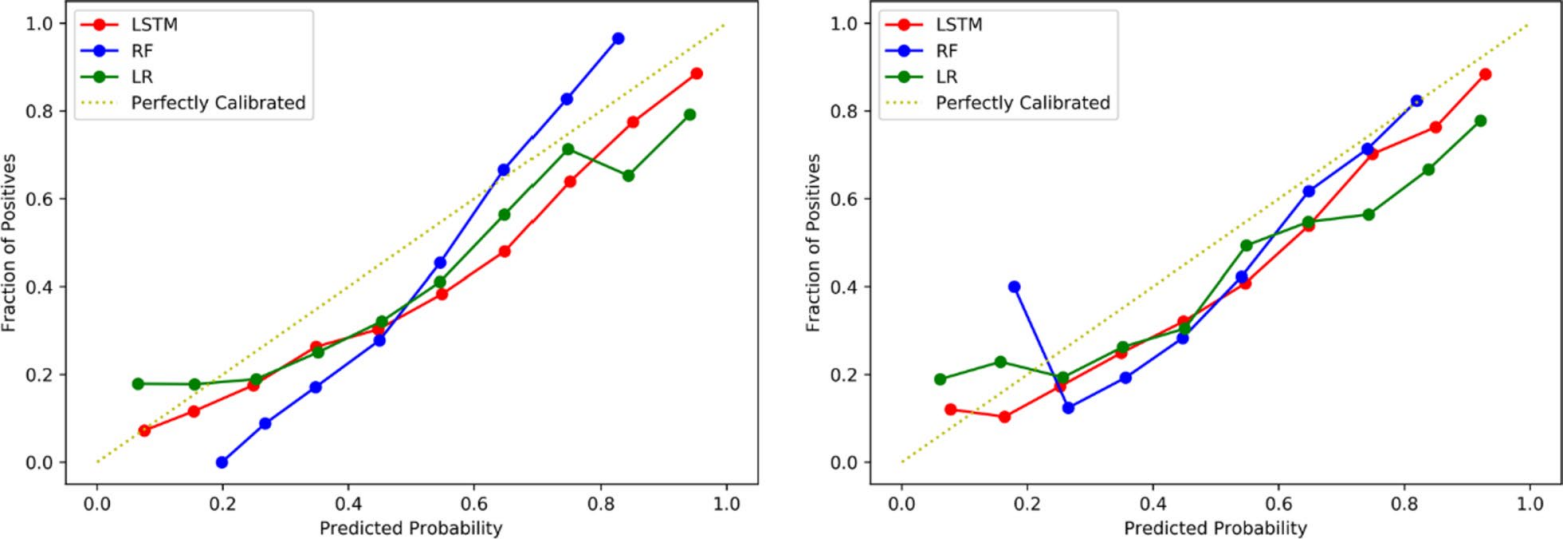
Calibration plots for each LSTM model: All patients (left) and patients admitted with respiratory disorders (right)

While the ability of the model to discriminate between patients at higher risk of having an event from those at lower risk is an important aspect, alone it is not sufficient. As such, we also consider model calibration, which measures how similar is the predicted absolute risk to the true observed risk in groups of patients. Poorly calibrated models will underestimate or overestimate the outcome of interest. As such we assess our model calibration by comparing predicted and observed risk of mortality at the whole patient population (mean calibration) as well as a subgroup of patients with respiratory disorders. As it can be seen in Fig. 5, all models achieved a very good calibration in predicting mortality risk for both the overall patient dataset and the subgroup of patients with respiratory disorders, even though models based on neural networks are typically poorly calibrated, as reported in the literature [26].

## Discussion

In this study a recurrent neural network-based model outperformed random forest and logistic regression models regarding outcome prognostication in a large cohort of mechanically ventilated, critically ill patients of the VENTILA study group when using six common ventilation parameters, extended by age and body mass index (BMI). Predictive performance was even increased when serum bilirubin (as a marker of intact liver function), serum creatinine (as a marker of unscathed kidney function), as well as pH and PaCO2 (as indicators of general metabolic and respiratory performance) were included.

pH, as the expression and common endpoint of both the metabolic and respiratory situation, was a relevant and potent predictor of outcome in both subgroups. In both subgroups, biomarkers for organ failure (bilirubin and creatinine, respectively) were relevant for the outcome of the patients evaluated. Also, in the subgroup of primarily pulmonary patients the classical ventilation parameters were not the singularly decisive parameters for the outcome, although the respiratory parameters were present in a relatively high granularity. Ultimately, however, this is not surprising, as even in critical patients with initial respiratory problems, a systemic cascade of inflammation and stress is set in motion that transcends the underlying pulmonological starting point.

Machine learning is a promising approach for multiple applications in modern medicine. Especially in critical care, studies with a vast number of patients are commonly not available for certain disease entities. Computer-based approaches expand our possibilities by facilitating the use of highly complex models with lots of different parameters and can therefore aid in complex clinical decision-making [1, 6, 27–29]. Of course, the cat bites its tail here, as ML benefits especially from large databases with significant patient numbers to exclude noisy data. Albeit the motto "the more, the better" certainly applies here, Shillan et al. were able to show that even for study sizes with 1000–10,000 individuals, satisfactory forecasts with AUCs around 0.83 can be achieved [30]. As demonstrated in our study, the use of additional variables can improve test performance and enable high prognostic accuracy in comparatively medium-sized patient collectives.

An association between the invasiveness of mechanical ventilation and/or oxygenation indices (especially the P/F value or Horowitz index) and mortality has been shown repeatedly in the past [14]. Different ventilatory parameters were found to be associated with mortality in previous studies. High driving pressures and tidal volumes, as well as low oxygenation indices were shown to be associated with higher mortality in mechanically ventilated patients, especially in individuals with acute respiratory distress syndrome (ARDS) in multiple previous studies [31–36]. It therefore seemed reasonable to combine various key ventilation parameters as mortality predictors in our ML model. Albeit mortality has decreased over time, higher age is a known predictor of worse outcome in mechanically ventilated patients, whereas low BMI-values were associated with decreased survival in the past [37, 38]. Hence it seemed rational to additionally incorporate such common, but outcome-relevant general patient characteristics into the model. As pulmonary performance is already indirectly covered by ventilation settings, inclusion of further non-ventilator associated, but vital organ function parameters seemed reasonable (namely serum creatinine and bilirubin). Although affected by ventilation strategies and certainly also kidney function, blood pH as a marker of metabolic integrity and partial pressure of carbon dioxide (paCO2) as an indicator of satisfactory ventilation were also included in our model and yielded an even higher predictive performance. It should be mentioned that several challenges could have affected the performance of the model, including the heterogeneity of data, given the multi-centre nature of the dataset spanning across diverse ICUs and countries; consequently, the heterogeneity of data collection protocols; and the averaging of ventilation parameters into a single daily value.

As already stated in the past, ML algorithms often lack transparency compared with conventional statistical analyses as they are not as reproducible for most external readers [30]. Nevertheless, considering the possibilities of our time, it seems reasonable trying to integrate them into our clinical practice in order to reflect our decisions in a sober light, based on different algorithms irrespective of gut feeling or other personal bias. Replacing medical specialists with artificial intelligence is certainly not the right way to go. Nevertheless, it seems rational, to reflect and analyse complex situations independently based on measurable (hard) criteria and therefore be able to make even better decisions for our patients in future.

Theoretically, and in this context, we consider our study to be theses-generating, an algorithm could not only serve to predict the outcome based on ventilation parameters. Rather, an attempt could be made to explore an optimal ventilation strategy on the basis of large data sets. An algorithm would also be conceivable, which as a (nearly) closed loop suggests ventilation parameters adapted to the individual situation, based on ventilation parameters, but also biomarkers and possibly other clinical and radiological variables, to the clinician. However, this is in any case beyond our data and analysis. Ultimately, our analysis can also be seen as a "word of caution" in this context: the high value of biomarkers (bilirubin and creatinine) underlines the relevance of a holistic approach. Therefore, despite all the enthusiasm for digital revolutions, it is important never to forget clinical relevance and practicability. In addition to these rather pragmatic considerations, ethical considerations regarding the use of AI in everyday clinical practice are also highly relevant. Algorithms, especially those that have a relatively direct influence on therapy, must be subjected to a critical, evidence-based evaluation—i.e. randomized clinical studies—in analogy to medical production.

## Limitations

Firstly, this is a retrospective study lacking a randomisation process, prospective screening, and inclusion of patients and a control group, therefore this study can only be thesis-generating. Secondly no specific protocol for the collection of predictive variables (e.g., specific timespan and/or clinical situations when to document MV parameters) was applied, which could further dispose of the study to selection bias as well as imputation strategy for the missing data. Lastly, it should be noted that LR and RF algorithms have not been designed to process the sequences directly, in contrast to LSTM, which may explain the difference in performance between these algorithms.

## Conclusion

The result of our analysis has shown that the RNN-based model demonstrated better performance than RF and LR in patients in mechanical ventilation and its subgroup admitted with respiratory disorders. However, it is necessary to validate our results in further studies. We speculate that a dataset with higher granularity—for example, more closely timed records—could lead to an even higher predictive power of AI. The next step would then be to develop algorithms that not only seek to predict outcome, but also suggest alternative ventilation parameters based on prior data, and, for example, seek to ensure even better use and application of evidence-based treatment strategies such as low driving pressure ventilation. If, in a next step, these suggestions—for example, in randomized trials—are associated with a survival benefit for our patients, then a further step would be the development of "closed loop" ventilation systems that seek to optimize the ventilation of critically ill patients on the basis of collected parameters and within evidence-based limits. However, this is currently to be classified as a theoretical possibility and we recall that the strict standards of evidence-based medicine must also be applied to AI—any algorithms must also prove their efficacy and safety in randomized trials. Medical ethics and legal issues must also be evaluated and discussed with all stakeholders at an early stage—how much control are physicians willing and able to relinquish, how much automated treatment are patients prepared to receive? These are interesting issues that currently remain unresolved and as a consequence we consider our study to be thesis generating.

## Data Availability

The data that support the findings of this study are available from the VENTILA Study Group, but restrictions apply to the availability of these data and so are not publicly available. Data are however available from the authors upon reasonable request.

## Abbreviations

AP: Average Precision
ARDS: Acute Respiratory Distress Syndrome
AUC: Area under the curve
AUPRC: Area under the precision recall curve
BMI: Body mass index
COPD: Chronic obstructive pulmonary disease
DL: Deep Learning
GPU: Graphical processing unit
IG: Integrated gradient
LoS: Length of stay
LR: Logistic Regression
LSTM: Long short-term memory
MCC: Matthews correlation coefficient
MIMIC: Medical Information Mart for Intensive Care
MV: Mechanical ventilation
NPV: Negative Predictive Value
PBW: Predicted body weight
PEEP: Positive end-expiratory pressure
PPV: Positive Predictive Value
RF: Random Forest
RNN: Recurrent Neural Network
ROC: Receiver Operating Characteristics
SAPS: Simplified Acute Physiology Score
